# Trajectory Learning Using HMM: Towards Surgical Robotics Implementation

**DOI:** 10.3390/s25113487

**Published:** 2025-05-31

**Authors:** Juliana Manrique-Cordoba, Carlos Martorell-Llobregat, Miguel Ángel de la Casa-Lillo, José María Sabater-Navarro

**Affiliations:** 1 Bioengineering Institute, Miguel Hernandez University of Elche, 03202 Elche, Spain; jmanrique@umh.es (J.M.-C.); mcasa@umh.es (M.Á.d.l.C.-L.); 2 Neurosurgery Unit, Hospital General de Elche, 03203 Elche, Spain; carlosmll1992@gmail.com

**Keywords:** hidden markov models, learning from demonstration, robotic trajectory learning, trajectory simplification, surgical robotics

## Abstract

Autonomy represents one of the most promising directions in the future development of surgical robotics, and Learning from Demonstration (LfD) is a key methodology for advancing technologies in this field. The proposed approach extends the classical Douglas–Peucker algorithm by incorporating multidimensional trajectory data, including both kinematic and dynamic information. This enhancement enables a more comprehensive representation of demonstrated trajectories, improving generalization in high-dimensional spaces. This representation allows clearer codification and interpretation of the information used in the learning process. A series of experiments were designed to validate this methodology. Motion data and force interaction data were collected, preprocessed, and used to train a hidden Markov model (HMM). Different experimental conditions were analyzed, comparing training using only motion data versus incorporating force interaction data. The results demonstrate that including interaction forces improves trajectory reconstruction accuracy, achieving a lower root mean squared error (RMSE) of 0.29 mm, compared to 0.44 mm for the model trained solely on motion data. These findings support the proposed method as an effective strategy for encoding, simplifying, and learning robotic trajectories in surgical applications.

## 1. Introduction

Surgical robotics has revolutionized medicine by enabling more precise and less invasive procedures, leading to faster patient recovery. This innovation integrates advanced engineering with surgical techniques, enhancing dexterity and reducing human error. A prominent example is the da Vinci Surgical System by Intuitive [1], widely used in minimally invasive surgeries, alongside systems such as Versius by CMR Surgical [2] or Hugo by Medtronic [3]. Robotic assistance has significantly advanced laparoscopic surgery by addressing the limitations of traditional techniques. Beyond facilitating complex procedures, these systems also create new opportunities for surgeon training and performance evaluation.

Modern surgical robots offer advanced capabilities such as three-dimensional visualization, flexible instrumentation, and motion scaling [4]. These features are crucial for optimizing precision in complex procedures like prostatectomies and hysterectomies, where accurate access and manipulation are essential. The integration of artificial intelligence and haptic feedback further enhances these tools, improving efficiency and accessibility. Moreover, robotic precision enables objective skill assessment, serving as a motion measurement tool to refine surgical training programs.

Currently, most commercially available surgical robots operate through transparent teleoperation, where the surgeon’s movements are directly replicated by the robotic tool with no additional feedback beyond the visual information provided by laparoscopic cameras. However, autonomy is increasingly recognized as one of the most promising directions for the advancement of surgical robotics, particularly in enabling the execution of simple, repetitive tasks to assist surgeons and reduce their workload [5,6,7,8]. To achieve this, Learning from Demonstration (LfD) and Programming by Demonstration (PbD) emerge as key methodologies for enabling robotic systems to acquire, generalize, and reproduce surgical trajectories effectively.

LfD plays a pivotal role in robotic trajectory planning, enabling robots to acquire complex motion patterns from expert demonstrations and adapt to dynamic environments. This approach encompasses both imitation learning and inverse reinforcement learning, optimizing trajectories while considering factors such as obstacle avoidance and safe tissue interaction [9]. In the context of surgical robotics, planned trajectories not only replicate precise movements but also prioritize patient safety by minimizing unintended tissue damage and optimizing surgical workflow [10]. A representative application is bone tissue milling, a procedure commonly required in various surgical interventions, where the applied force significantly influences the integrity of the bone itself as well as surrounding anatomical structures. In such tasks, LfD enables the encoding and reproduction of force-sensitive motions demonstrated by experts, promoting safety and effectiveness in robotic execution. At the trajectory level, skill learning involves modeling a demonstrated set of trajectories and retrieving a generalized representation suitable for robotic reproduction. To effectively capture the inherent variability in human-demonstrated motions, many studies have employed probabilistic representations of recorded data from multiple demonstrations of the same skill [11].

Several approaches have been widely used for modeling demonstrated tasks at a trajectory level, including hidden Markov models (HMMs) [12], Gaussian mixture models (GMMs) [13], and probabilistic movement primitives (ProMPs) [14,15]. Among these, HMMs have been extensively applied in the field of Programming by Demonstration (PbD) due to their robustness in handling spatiotemporal variations in sequential data [16]. Within the LfD framework, expert-demonstrated trajectories are effectively modeled using HMMs, allowing for the capture of both spatial and temporal variations in movement patterns. Well-trained HMM parameters facilitate the reconstruction of optimal state sequences and the generation of new, generalized trajectories, ensuring adaptability and precision in robotic motion planning [9].

Furthermore, trajectory simplification techniques play a crucial role in optimizing training data, improving computational efficiency while maintaining the integrity of demonstrated movements. Methods such as Douglas–Peucker [17] and Visvalingam–Whyatt [18] reduce redundant information while preserving essential characteristics. By simplifying high-dimensional trajectory data, these techniques contribute to reducing the complexity of robot learning models, allowing for faster processing and better generalization.

The current challenge is addressed using models that rely solely on robotic motion data, such as position and velocity [7,9], while ignoring other critical factors, including interaction forces with tissues and spatial relationships between different surgical tools. This limitation can lead to imprecise learning and the generation of suboptimal trajectories, reducing overall system efficiency.

To overcome these constraints, a more comprehensive approach is required, that simultaneously integrates multiple data modalities, such as position, velocity, acceleration, and/or force. By normalizing this multidimensional information, it is possible to achieve a balance between computational efficiency and trajectory precision, ultimately enhancing the adaptability and performance of robotic systems.

This article presents a novel approach for incorporating multiple dimensions of information, including both motion-related parameters (position and velocity) and interaction forces within the workspace (forces and torques), into a Learning from Demonstration (LfD) framework. Unlike previous works that rely solely on kinematic data [7,9], our methodology leverages the inclusion of force information to improve the accuracy and adaptability of learned trajectories. This is particularly relevant in tasks where force plays a critical role, such as bone tissue milling, where excessive or insufficient applied force can compromise safety or performance. The proposed approach combines trajectory simplification, normalization, and encoding using a hidden Markov model (HMM), and enables the generation of new trajectories that reflect both geometric patterns and dynamic interaction with the environment. While this study focuses on surgical robotics, the methodology is applicable to other domains requiring context-aware and force-sensitive robotic behavior, such as industrial automation and collaborative robotics.

The structure of the paper is as follows: Section 2 provides details on the algorithm used. First, the data acquisition procedure is described, followed by the preprocessing steps applied to the collected signals, including filtering, normalization, and simplification. Next, the methodology for generating the necessary data to train the implemented HMM is outlined. The details regarding the methodology applied for the training algorithm can be found in [19]. Finally, the decoding process is explained, which converts the predicted sequence back to its original units.

The results of the proposed experiments are presented in Section 3, where a comparative analysis of HMM training is conducted using different datasets for the same procedure. One experiment considers only motion data, while another includes information related to the interaction with the workspace environment. Finally, Section 4 summarizes the key findings of this study, discusses potential improvements to the proposed methodology, and outlines future research directions for this line of work.

## 2. Materials and Methods

**Figure 1:**
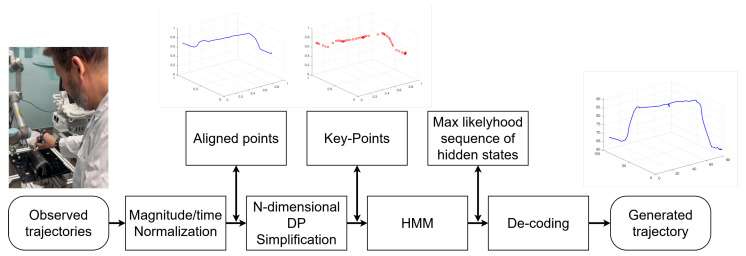
Flowchart of the proposed framework.

A schematic diagram depicting the data processing flow for the proposed approach is shown in Figure 1, illustrating a systematic method for generating optimized robotic trajectories from sensed multidimensional data (i.e., kinematic information such as position, velocity, and/or acceleration and dynamic information, such as interaction forces, torques, and/or tissue stiffness). Initially, raw trajectories are captured and undergo magnitude/time normalization to ensure consistency. The data is then aligned spatially and temporally, followed by *N*-dimensional Douglas–Peucker simplification to reduce complexity while maintaining essential characteristics. Key-points extraction identifies the most relevant features, which are then processed using a hidden Markov model (HMM) to estimate the most likely sequence of hidden states. The trajectory is then decoded and reconstructed, resulting in a generated multidimensional trajectory that accurately performs the learned procedure. A detailed description of the approach follows in this section.

### 2.1. Data Acquisition

**Figure 2:**
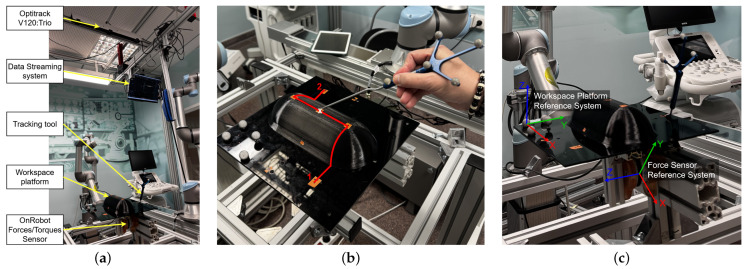
(**a**) Experimental setup. (**b**) Visualization of the demonstrated trajectory to be executed using the tracking tool. (**c**) Reference frame configuration of the force sensor.

Figure 2a illustrates the experimental setup used for data collection. A “Workspace Platform” has been installed as the primary surface for trajectory execution. This platform is instrumented to maximize data acquisition. It is mounted on a force sensor (OnRobot A/S. Odense, Denmark) [20], which continuously records variations in forces and torques exerted during trajectory execution. Additionally, an OptiTrack V120:Trio (NaturalPoint, Inc., Corvallis, OR, USA) optical tracking system [21] is positioned above the setup to accurately capture the position and orientation of the tool performing the trajectory. The upper section of the image shows the data acquisition system, which transmits the recorded information in real time through a communication network for subsequent storage and processing. This configuration enables high-resolution monitoring of the interaction forces and precise spatial tracking, ensuring accurate and reliable trajectory data.

On the other hand, Figure 2b provides a detailed view of the workspace platform, which includes a designated obstacle. The predefined trajectory performed by the users is highlighted in red, starting at Point 1. The movement begins with an upward motion along the obstacle in a straight line, followed by a perpendicular transition across the obstacle, and finally reaching Point 2 in a straight-line descent. Participants are instructed to maintain continuous contact with the platform throughout the movement. Additionally, as they pass through the white “X” marked in the figure, they are required to apply slight pressure on the platform. This interaction introduces controlled force variations into the dataset, allowing for a more detailed analysis of user-applied forces along the trajectory.

Finally, Figure 2c presents the configuration of the force sensor’s reference system, which is used to register the interaction forces exerted on the platform during trajectory execution. The force sensor provides real-time measurements along multiple axes, enabling a precise quantification of contact forces and torques. This information is essential for analyzing force distribution patterns and refining robotic models based on human demonstrations.

A total of 10 users were instructed to perform the trajectory three times, resulting in a dataset of 30 recorded samples. For each trial, a sequence of trajectory points was collected, capturing detailed motion and force-related information. Each point in the trajectory dataset stores a timestamp along with 12-dimensional motion and interaction data, structured as follows: three positional coordinates of the tool’s end effector (*X*, *Y*, *Z*), three velocity components (Vx, Vy, Vz), three force measurements (Fx, Fy, Fz), and three torque components (Tx, Ty, Tz). This comprehensive dataset enables a detailed analysis of both kinematic and dynamic aspects of the demonstrated procedure, allowing for a more accurate representation of the interaction forces and torques exerted throughout the trajectory execution.

### 2.2. Signal Preprocessing

Once the raw experimental data is collected, a preprocessing stage is performed to enhance data quality before further analysis. This process includes signal filtering to remove noise and normalization in both temporal and magnitude domains. These steps are essential to ensure that the data is properly conditioned for subsequent processing using the *N*-dimensional simplification algorithm.

Regarding temporal normalization, only the relevant segment of the trajectory is retained, starting from the moment the user makes contact with the workspace platform at Point 1 until they reach Point 2 (Figure 2b) and end contact with the platform. To standardize trajectory execution across different trials, the time axis is interpolated so that the temporal values correspond to the percentage of trajectory completion, ranging from 0% to 100%. This transformation allows for a consistent representation of temporal evolution across multiple demonstrations, making the dataset more suitable for learning-based models and trajectory generalization.

#### 2.2.1. Filtering

The raw position signal exhibits high precision; therefore, the only preprocessing step applied is a reference system transformation to align the data with the workspace platform coordinates. This ensures consistency across all recorded trajectories and facilitates further analysis within a unified spatial framework.

Conversely, force signals are inherently more susceptible to noise due to the relatively low magnitudes of the forces and torques exerted during trajectory execution. To mitigate this issue, a moving average filter is applied to smooth the force measurements and reduce high-frequency fluctuations. This filtering process enhances the reliability of the recorded force data while preserving the essential characteristics of the user’s interaction forces.

#### 2.2.2. Normalization

Since the trajectory dataset is multidimensional, each trajectory point contains several magnitudes, such as position, velocity, and interaction forces. To ensure consistent scaling across variables and avoid distortions due to differing units or ranges, a normalization and scaling procedure is applied independently to each magnitude.

The raw data is organized as a set of matrices, one for each type of signal (e.g., position, velocity, force), with each matrix containing time-series data from all demonstrations. For each of these matrices, a min-max normalization is applied, scaling all values to the [0,1] range based on the global minimum and maximum of the respective signal. This preserves the internal variability of each signal while eliminating offsets and unit inconsistencies.

After normalization, each signal is further scaled by a predefined weight coefficient that allows for adjusting the relative contribution of each variable in later processing steps. For example, if forces are considered more relevant for the learning task, they may be given a higher weight compared to position or velocity. This weighted normalization results in a balanced, dimensionally consistent dataset suitable for subsequent stages, such as trajectory simplification and learning-based modeling.

**Figure 3:**
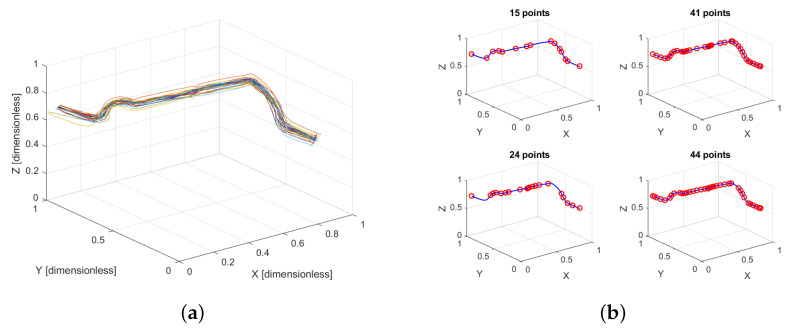
(**a**) Normalized demonstrated trajectories performed by the users, where each color represents a different user. (**b**) *N*-dimensional simplification applied to the same trajectory, considering different sets of dimensions: (**top-left**) 3 dimensions (position only), (**top-right**) 6 dimensions (position and velocity), (**bottom-left**) 9 dimensions (position, velocity, and force), and (**bottom-right**) 12 dimensions (position, velocity, force, and torque).

Figure 3a shows the normalized trajectories performed by different users, highlighting how the procedure unifies the scale across magnitudes while retaining the essential shape and variability of the original data.

### 2.3. Key-Point Simplification

A method is required to represent key trajectory points in a multi-dimensional space while preserving their temporal association. The modified Douglas–Peucker reduction algorithm, as presented in [19], is implemented to simplify trajectories while preserving key motion characteristics. The algorithm begins with a set of sampled points organized in a matrix *R*, where each row contains the position, velocity, force, and other trajectory-related parameters. The process starts by evaluating the Euclidean distance d(n) between the first and last points of the trajectory:

(1) $$d\left(n\right)=\sqrt{{\left(F_{n}-F_{1}\right)}^{2}+...+{\left(Y_{n}-Y_{1}\right)}^{2}+{\left(X_{n}-X_{1}\right)}^{2}}$$

where *F* corresponds to the applied force, *Y* and *X* represent the position coordinates along their respective axes, and so on; the notations *n* and 1 denote the indices of the last and first points in the trajectory, respectively. If this distance is below a computational threshold, the trajectory is considered sufficiently simplified, and only the endpoints are retained. Otherwise, the maximum orthogonal distance dp(k) from any intermediate point to the line connecting the first and last points is computed. The orthogonal distance is determined using the following equation:

(2) $$dp\left(k\right)=\frac{∥(P-A)\times (B-A)∥}{∥B-A∥}$$

where:

- × represents the cross product in 2D/3D or its generalization to higher dimensions using an orthogonal projection.
- ∥·∥ denotes the Euclidean norm.
- *A* and *B* represent the first and last points of the evaluated segment, respectively.
- *P* is the point whose orthogonal distance to the line segment $\bar{AB}$ is being calculated.

If the maximum orthogonal distance exceeds a predefined tolerance ε, the trajectory is recursively divided into two segments, and the same process is applied iteratively to each sub-segment. Otherwise, the trajectory is reduced to the key points that best approximate the original path while minimizing data redundancy. For further details on the implementation of this algorithm, refer to the study published by Manrique et al. [19]

The performance of the proposed approach is evaluated by applying the *N*-dimensional simplification algorithm to a representative trajectory performed by a single participant. The original trajectory consists of 53 points. As a preprocessing step, each dimension (position, velocity, force, and torque) is independently normalized, resulting in dimensionless data that preserve the relative variation across magnitudes. The simplification algorithm is then applied using different combinations of input dimensions, while maintaining a constant tolerance ε.

Figure 3b shows the resulting simplified trajectories, where the number of retained points reflects the influence of the selected dimensions:

- Top-left (15 points): Simplification using only the 3D position components (X, Y, Z). This yields a sparse representation focused on preserving geometric shape, with fewer points required.
- Top-right (41 points): Simplification using position and velocity (6 dimensions). The inclusion of velocity captures temporal dynamics, leading to a denser point distribution in regions with greater speed variation.
- Bottom-left (24 points): Simplification using position, velocity, and force (9 dimensions). Although the force signal varies less than velocity, it influences key-point selection, especially in segments where contact with the obstacle occurs, preserving important interaction details.
- Bottom-right (44 points): Simplification using all 12 dimensions (position, velocity, force, and torque). Torque data add additional complexity, resulting in the highest number of retained points and a finer representation of the trajectory’s dynamic characteristics.

This comparison highlights how incorporating multidimensional data enhances the representation of critical motion and interaction features, allowing the simplification algorithm to retain more detail where necessary.

### 2.4. Codebook Formation

Once the trajectory data is discretized based on user-defined resolution parameters, it is subsequently encoded into a codebook. A codebook, in this context, refers to a Cartesian lookup table where each possible combination of discretized parameter values is mapped to a unique integer code. This encoding process allows efficient storage and retrieval of trajectory information, facilitating rapid execution while maintaining the required precision for the task.

The resolution used for discretization is chosen according to the application requirements (e.g., the tip size of a drill or the area of effect of a cauterizer), and it directly affects the level of detail retained in the trajectory. For example, positional data might be discretized into 20 intervals, while force signals are grouped into 10, based on their variability and relevance.

The complete implementation of this process, including interpolation, quantization, and codebook indexing, is described in detail in our previous work [19], which includes all necessary mathematical formulations and diagrams. In the present work, this procedure is adopted directly to build the observation sequence for the hidden Markov model (HMM), enabling accurate modeling of motion behaviors across multiple users.

### 2.5. Hidden Markov Model

The HMM is implemented by defining two key probability matrices: the transition matrix (A) and the emission matrix (B). The transition matrix *A*, of size m×m, represents the probability of transitioning from one state to another, where each element $A_{\left(i,j\right)}$ denotes the probability of moving from state *i* to state *j*. The emission matrix *B*, with dimensions $m\times N_{t}$, defines the probability of emitting an observable symbol from a given state, where $B_{\left(i,k\right)}$ represents the probability of emitting symbol *k* from state *i*.

Here, *m* represents the number of hidden states in the model, which corresponds to the total number of possible combinations defined in the codebook. Meanwhile, $N_{t}$ corresponds to the maximum number of discrete values that the observable variables can take, determined by the resolution used to discretize the trajectory execution time. These matrices are estimated in MATLAB 2024b using the *hmmestimate* function, which computes maximum likelihood estimates of transition and emission probabilities based on observed sequences and their corresponding hidden states [22].

Once the matrices have been trained, they are used to determine the most likely sequence of hidden states that best explains the observed emissions. This is achieved through the Viterbi algorithm, which maximizes the likelihood of the state sequence given the model parameters. The algorithm is implemented using the *hmmviterbi* function in MATLAB 2024b, which initializes the model in state 1 at step 0 before the first emission.

### 2.6. De-Codification

The HMM implementation involves two key stages: training and decoding. During training, the model learns the probabilistic relationships between hidden states and observed variables using a predefined codebook (CB), which discretizes the data into specific groups depending on the desired resolution. Before preprocessing, the maximum ($max_{i}$) and minimum ($min_{i}$) values for each dataset are recorded to ensure consistency in the discretization process. Once the HMM generates a sequence of predicted discrete states, a decoding step is required to map these states back to their original physical range.

The decoding process applies a rescaling equation to convert the discrete predictions into continuous values that align with the original dataset. The transformation is performed using the following:

(3) $$p_{real}=min_{i}+\frac{\left(p_{discrete}+0.5\right)\left(max_{i}-min_{i}\right)}{CB_{i}}$$

where $p_{real}$ represents the rescaled value in the original range, $p_{discrete}$ is the discrete state predicted by the HMM, $CB_{i}$ is the total number of discrete groups, and $max_{i}$ and $min_{i}$ define the bounds of the original dataset. This approach ensures that the reconstructed trajectory maintains consistency with the initial magnitudes of the variables, allowing the robotic system to execute learned movements.

## 3. Results and Discussion

The following section presents a comprehensive analysis of the trajectories generated by incorporating different sets of trajectory data into the model training process. This study investigates how the trajectory generation process is influenced by varying sequences of observable variables, including position, velocity, force, and torque. Understanding these variations is crucial for optimizing the learning process and improving the model’s ability to accurately generate a multidimensional trajectory learned from the procedure.

As previously mentioned, a total of 30 trajectories were acquired for this study. On average, each acquired trajectory consists of 73 points, with the shortest trajectory containing 42 points and the longest one comprising 102 points. After applying the *N*-dimensional simplification process, the shortest trajectory is reduced to 18 points, while the longest one is simplified to 88 points, resulting in an average of 53 points per trajectory.

The selection of the simplification tolerance values (ε=0.01 and ε=0.005) and discretization resolutions (e.g., 20 groups for velocity data) was based on an empirical tuning process. The tolerance values were chosen to retain critical motion features while effectively reducing the size of the trajectory data. It is important to note that the primary goal of trajectory simplification is to reduce the computational cost associated with processing the data using HMMs. In principle, the full dataset could be used without simplification; however, this would significantly increase computational requirements. Preliminary tests indicated that variations of 20% in these parameters did not substantially affect trajectory reconstruction accuracy.

Given this distribution, a trajectory consisting of 53 points after simplification is selected as the test trajectory, as it represents the average length of the dataset. Therefore, out of the 30 recorded trials, 29 are initially used to train the HMM model, and one trajectory—selected based on its average length after simplification—is used to extract the observable sequence for model validation. Although this trajectory is not necessarily from the most experienced participant, its representative characteristics make it a suitable reference.

To generate the trajectory from the trained HMM, the normalized time vector of the test trajectory is utilized. This vector is discretized into $N_{t}$ groups of possible observable variables, and a sequence is defined according to the division of these groups. This sequence is then provided to the model to obtain the corresponding hidden states, which represent the possible combinations (form the codebook) associated with the given sequence.

To further evaluate the generalization capability of the model, a second experiment is conducted in which all 30 trajectories are used for training. In this case, the observable variable sequence is generated digitally by uniformly subdividing a normalized time vector based on the average trajectory length.

### 3.1. Model Training Using Only Motion Data

**Figure 4:**
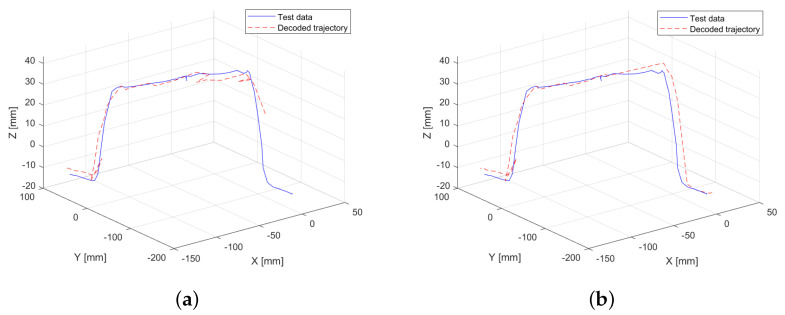
Comparison between the validation trajectory (test data) and the trajectory generated by the HMM trained with motion data only (decoded trajectory). (**a**) Resolution of 10 units for all velocity axes. (**b**) Resolution of 20 units for velocity in the x- and y-axes, and 10 units for the z-axis.

Initially, the model is trained using a conventional approach, relying solely on motion data, specifically position and velocity. The resolution defined for grouping in the codebook is set to 100 units for position and 10 units for velocity, assigning greater importance to positional data. The results of this experiment are illustrated in Figure 4a, where discontinuities can be observed along the trajectory. These discontinuities result in a root mean squared error (RMSE) of 0.44 mm between the test data and the decoded generated trajectory.

To further analyze the impact of velocity resolution, the experiment is repeated with an increased resolution for velocity values in the x- and y-axes, as these exhibit the most significant variations. The objective is to evaluate how this adjustment influences the predicted trajectory. As shown in Figure 4b, no apparent repetitive discontinuities are observed in the generated trajectory. This highlights that the learning process is directly influenced by the preprocessing parameters applied to the training data. As a result of this experiment, the RMSE is reduced to 0.34 mm, representing a total reduction of 22.73% demonstrating the effect of velocity resolution on trajectory accuracy.

It is important to note that while modifying the velocity resolution improves the performance of trajectory generation, it does not provide sufficient information to accurately define the point at which pressure was applied to the obstacle. As a result, the model continues the trajectory without explicitly identifying this interaction.

### 3.2. Interaction Forces

As previously mentioned, Figure 2c illustrates the reference frame configuration of the force sensor. From this configuration, it can be inferred that significant variations in the recorded forces will be observed along the x- and y-axes, whereas the z-axis, being parallel to the platform surface, will exhibit minimal variations. Based on this analysis, a resolution of 20 units is assigned to the x- and y-axes, while a resolution of 5 units is defined for the z-axis to reflect the expected force distribution more accurately.

**Figure 5:**
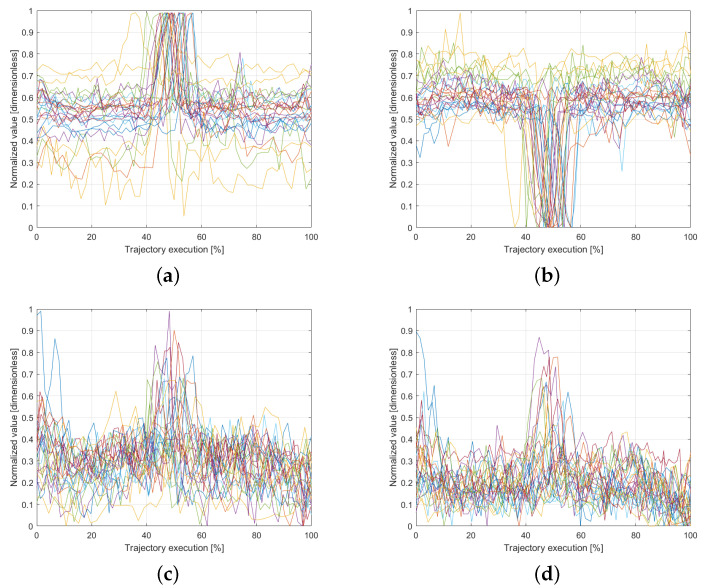
Normalized interaction force and torque data recorded during task execution. Each colored line represents the data from a different user. (**a**) Force in the *x*-axis. (**b**) Force in the *y*-axis. (**c**) Torque in the *x*-axis. (**d**) Torque in the *y*-axis.

Figure 5 presents the force and torque readings recorded along the x- and y-axes for all 30 trials. A noticeable variation is observed around the midpoint of the trajectory execution, coinciding with the moment when users were instructed to apply pressure on the platform. This pattern highlights a key interaction point, providing valuable information for training the model with interaction-based data. The ability to capture such variations ensures that the system can learn not only from motion dynamics but also from physical interactions within the workspace, improving the accuracy of trajectory generation in response to external forces.

### 3.3. Model Training Using Motion Data and Linear Forces

In the following experiment, the performance of the HMM is evaluated when trained with motion data along with the interaction forces exerted on the platform, as recorded by the OnRobot force sensor in each axis. This setup does not include torque information, focusing solely on the contribution of linear force data to trajectory generation.

**Figure 6:**
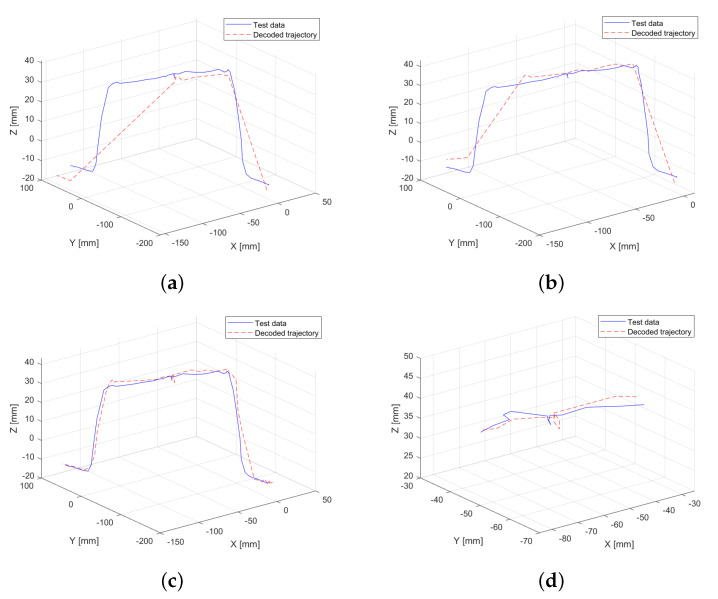
Comparison between the validation trajectory (test data) and the trajectory generated by the HMM trained with motion and force interaction data (decoded trajectory). (**a**) Model trained with motion and force data, using a simplification tolerance of 0.01 (dimensionless). (**b**) Model trained with motion and force data, using a simplification tolerance of 0.005 (dimensionless). (**c**) Model trained with motion, force, and torque data, using a simplification tolerance of 0.01 (dimensionless). (**d**) Zoom-in on a critical trajectory segment (midpoint) from the model trained using motion, force, and torque data, with a simplification tolerance of 0.01 (dimensionless).

Figure 6a,b summarize the results. First, the model is trained after simplifying the trajectory using a tolerance of 0.01 (Figure 6a) applied to the normalized data (scaled to 1). The inclusion of an additional magnitude (three extra dimensions) impacts key-point selection, leading to information loss at the endpoint of the obstacle. Consequently, the RMSE is 0.46 mm. To mitigate this loss, the experiment is repeated with a lower tolerance of 0.005, maintaining normalization to 1, which increases the number of retained key points. This adjustment results in improved trajectory fitting (Figure 6b), reducing the RMSE to 0.31 mm. Adjustments to the tolerance value resulted in a reduction of 31.62% in these experiments. Overall, this represents a total reduction of 29.55% when comparing the first experiment, conducted using only motion data, with the experiment that includes linear force data.

### 3.4. Model Training Using Motion Data and Full Force Interaction Data

In this experiment, the model is trained using all available data, incorporating both motion data (position and velocity) and full interaction data (force and torque in all axes). Similar to the previous experiment, a higher resolution is assigned to the torques in the x- and y-axes (20 units), while a lower resolution is maintained for the z-axis (5 units) to account for its reduced variability.

The result of this experiment is shown in Figure 6c, where the decoded HMM generated trajectory closely resembles the original one. The inclusion of three additional dimensions has once again influenced the *N*-dimensional simplification process, preventing the loss of key points at the end of the trajectory. Furthermore, the model successfully captures the effect of user-applied pressure at the center of the obstacle (Figure 6d), preserving more key points in regions where significant variations in force and torque are detected. This demonstrates that integrating complete interaction data improves the accuracy of trajectory reconstruction, particularly in sections where external forces play a critical role. As a result, the RMSE for this experiment decreases to 0.29 mm, representing a significant reduction of 34.09% compared to the first experiment conducted.

### 3.5. Digitally Generated Observable Variables Sequence

**Figure 7:**
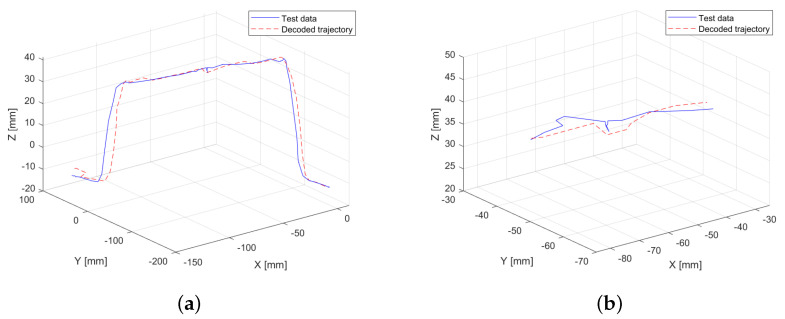
Comparison between the “ideal” trajectory (test data) and the HMM-generated trajectory from the digitally created observable variables sequence (decoded trajectory). (**a**) Overall comparison of both trajectories. (**b**) Close-up of the trajectory midpoint, highlighting the subtle force application captured by the decoded trajectory.

The previously conducted experiments involve a model trained using multidimensional data from 29 procedures performed by different users, while an “ideal” trajectory was selected to extract the sequence of observable variables and compare it with the trajectory generated by the learning model. To assess the robustness of the learning model, a retraining process is carried out using all 30 available multidimensional trajectory data. In this case, the sequence of observable variables is generated digitally. The average length of the simplified sequences is calculated, and a vector of “artificial” observable variables is created by uniformly subdividing the data into the predefined number of groups $N_{t}$. The results are shown in Figure 7a, where the trajectory generated from an “artificial” sequence of observable variables is represented by the red dashed line. This trajectory is compared with the “ideal” trajectory from the previous experiment (blue line), providing a visual reference of one of the executions performed by a user.

Unlike extracting the sequence of observable variables from a real trajectory, the digitally generated sequence does not allow for variable point density in specific phases of the motion. Nevertheless, the resulting trajectory closely approximates the ideal execution, even reproducing a subtle force application observed at the midpoint (Figure 7b). This outcome highlights the robustness of the learning model. The RMSE when comparing the generated trajectory to the real one is 0.21 mm, which represents a 52.27% reduction in error compared to models trained using only motion data.

## 4. Conclusions

Previous studies in the state of the art illustrate methodologies for trajectory learning in robot programming by demonstration, where HMMs are trained based on key-point identification derived from changes in position and velocity within demonstrated trajectories [7,9,11]. The main contribution of this article is to present an alternative learning approach compared to existing studies, incorporating both kinematic information, such as position and velocity, and dynamic information, such as interaction forces and torques. This approach enables the inclusion of multidimensional data in the training of LfD models, specifically in the implementation of HMMs.

A controlled experiment was designed to ensure data variability across different dimensions, as shown in Figure 2b. A workspace platform was set up with various sensors to capture both the motion data of a tool within the environment and the interaction forces exerted on the platform. Ten users participated in the experiment, each repeating the task three times, resulting in a dataset of 30 recorded trials. Each dataset contains three positional coordinates of the tool’s end effector (*X*, *Y*, *Z*), three velocity components (Vx, Vy, Vz), three force measurements (Fx, Fy, Fz), and three torque components (Tx, Ty, Tz).

The acquired multidimensional trajectories undergo filtering and magnitude-based normalization as preprocessing steps before being simplified using the *N*-dimensional modification of the Douglas–Peucker algorithm. The simplified trajectories are then encoded according to a predefined resolution for each set of magnitudes and subsequently used to train the learning model (HMM). Further details on the applied methodology can be found in [19].

From the obtained results, it can be highlighted that the proposed methodology enables a comparative analysis of training the same model with identical trajectories and resolution while incorporating different datasets in each experiment. The tested configurations range from using only motion data to fully integrating dynamic interaction data between the tool and the workspace platform.

The results from training exclusively with motion data indicate that the resolution selection in the codebook directly influences trajectory generation. Specifically, a reduction of 22.73% was obtained when generating the trajectory with a resolution of 20 units for the velocity magnitude, compared to using a velocity resolution of 10 units. It is important to emphasize that, even with the increased resolution in the velocity magnitude, the generated trajectory did not capture the positional variation associated with the force applied at the midpoint of the trajectory. This effect becomes evident in subsequent experiments.

Regarding the experiments that include both linear force data and full force interaction data, a decrease in RMSE is observed. When simplifying the trajectory using only motion and linear force data, a loss of information occurs in the second half of the trajectory. However, this error is corrected when incorporating full force interaction data, resulting in the lowest RMSE among all real-data experiments, with a reduction of 34.09% compared to the first experiment and the experiment with full training information. When visually comparing this result with the test trajectory in Figure 6c, a high degree of similarity is evident in the generated trajectory. The model successfully captures key trajectory features, including the movement associated with the force exerted at the midpoint of the trajectory, further validating the effectiveness of the proposed approach.

All experiments were conducted using the same sequence of observable variables. Thus, even when modifying the resolution of training parameters and the simplification tolerance, a more accurate generated trajectory was obtained when incorporating a greater amount of force interaction data.

Finally, to evaluate the robustness of the model, a final experiment is conducted in which the model is trained using the complete dataset, while the sequence of observable variables is generated digitally. This sequence is constructed based on the average length of the training observable variable sequences and the number of groups $N_{t}$ into which they are discretized. The results of this experiment are shown in Figure 7, where the learned trajectory closely resembles the test trajectory used for visual comparison. This experiment shows a significant error reduction of 52.27% compared to the trajectories generated with the model trained exclusively on motion data and using observable variables obtained from a real trajectory. Such a result is highly acceptable, considering that the trajectory does not account for the distribution of key points, as they have been uniformly defined. These findings highlight the robustness of the model when handling observable sequences that differ from those used during training.

The inclusion of both force and torque data provides valuable insights into user interaction dynamics, facilitating the development of more advanced robotic models capable of learning from human demonstrations. Moreover, the structured time-series nature of the dataset allows for further processing using probabilistic modeling techniques, such as HMMs, ensuring that variations in execution can be captured and generalized. This capability is particularly relevant for applications in robotic-assisted surgery and teleoperation, where learning from demonstrations must account for both motion characteristics and force-based interactions to enhance trajectory accuracy and task repeatability.

Future work will focus on several directions. First, the integration of geometric and dynamic relationships between different tools, as well as their simultaneous interaction with surrounding tissues, will be explored. Second, implementing closed-loop force and position control in robotic manipulators is planned to ensure safe and accurate reproduction of the acquired trajectory data. Third, broader validation of the proposed methodology across a wider variety of tasks and trajectories will be conducted to assess its adaptability and robustness beyond the specific case studies presented in this work. This includes evaluating how the learned models perform under different surgical scenarios and adapting the generated trajectories to account for anatomical variability between patients, which will require surgical registration and real-time trajectory adaptation mechanisms. Additionally, the influence of surgical skill level on model training and validation will be investigated to understand how demonstrations from expert versus novice users affect learning outcomes. A formal sensitivity analysis is also considered as future work to further assess the robustness of the selected parameter values. Finally, future work will include replicating the proposed methodology using alternative trajectory learning models such as Gaussian mixture models (GMMs) and probabilistic movement primitives (ProMPs), allowing for a systematic comparison across methods that rely on different modeling assumptions and input modalities.

## 5. Patents

The results presented in this article are documented and protected under the patent with File No. EP25382177, filed on 28 February 2025.

## Data Availability

Data is provided within the manuscript and it can be downloaded at https://medicalrobotics.umh.es/datarepository/.
